# A Chemo-Genomic Approach Identifies Diverse Epigenetic Therapeutic Vulnerabilities in MYCN-Amplified Neuroblastoma

**DOI:** 10.3389/fcell.2021.612518

**Published:** 2021-04-21

**Authors:** Aleksandar Krstic, Anja Konietzny, Melinda Halasz, Peter Cain, Udo Oppermann, Walter Kolch, David J. Duffy

**Affiliations:** ^1^Systems Biology Ireland and Precision Oncology Ireland, School of Medicine, University College Dublin, Dublin, Ireland; ^2^Centre for Molecular Neurobiology Hamburg (ZMNH), Emmy-Noether Group “Neuronal Protein Transport”, University Medical Centre Hamburg-Eppendorf (UKE), Hamburg, Germany; ^3^Botnar Research Centre, NIHR Oxford Biomedical Research Unit, Institute of Musculoskeletal Sciences, University of Oxford, Oxford, United Kingdom; ^4^Centre for Medicines Discovery, University of Oxford, Oxford, United Kingdom; ^5^Conway Institute of Biomolecular & Biomedical Research, University College Dublin, Dublin, Ireland; ^6^The Whitney Laboratory for Marine Bioscience and Sea Turtle Hospital, University of Florida, St. Augustine, FL, United States; ^7^Department of Biology, University of Florida, Gainesville, FL, United States

**Keywords:** cAMP-response-element-binding [CREB] protein (known as CBP or CREBBP), E1A Binding Protein P300 (known as EP300 or p300), SMARCA, epigenetic regulation, cancer, precision medicine, zebrafish

## Abstract

Although a rare disease, neuroblastoma accounts for the highest proportion of childhood cancer deaths. There is a lack of recurrent somatic mutations in neuroblastoma embryonal tumours, suggesting a possible role for epigenetic alterations in driving this cancer. While an increasing number of reports suggest an association of MYCN with epigenetic machinery, the mechanisms of these interactions are poorly understood in the neuroblastoma setting. Utilising chemo-genomic approaches we revealed global MYCN-epigenetic interactions and identified numerous epigenetic proteins as MYCN targets. The epigenetic regulators HDAC2, CBX8 and CBP (CREBBP) were all MYCN target genes and also putative MYCN interactors. MYCN-related epigenetic genes included SMARCs, HDACs, SMYDs, BRDs and CREBBP. Expression levels of the majority of MYCN-related epigenetic genes showed predictive ability for neuroblastoma patient outcome. Furthermore, a compound library screen targeting epigenetic proteins revealed broad susceptibility of neuroblastoma cells to all classes of epigenetic regulators, belonging to families of bromodomains, HDACs, HATs, histone methyltransferases, DNA methyltransferases and lysin demethylases. Ninety-six percent of the compounds reduced MYCN-amplified neuroblastoma cell viability. We show that the C646 (CBP-bromodomain targeting compound) exhibits switch-like temporal and dose response behaviour and is effective at reducing neuroblastoma viability. Responsiveness correlates with MYCN expression, with MYCN-amplified cells being more susceptible to C646 treatment. Thus, exploiting the broad vulnerability of neuroblastoma cells to epigenetic targeting compounds represents an exciting strategy in neuroblastoma treatment, particularly for high-risk MYCN-amplified tumours.

## Introduction

Epigenetics is defined as non-DNA encoded heritable modifications, which result in altered gene expression levels (Jin et al., 2011). Increasingly, epigenetics modifications have functional roles in human cancers (Sharma et al., 2010; Wainwright and Scaffidi, 2017). Indeed it has been postulated that neuroblastoma tumours, which lack recurrent somatic mutations (Pugh et al., 2013), may be driven by aberrant epigenetic signalling (Domingo-Fernandez et al., 2013; Veschi et al., 2017; Durinck and Speleman, 2018; Ram Kumar and Schor, 2018; Upton et al., 2020). Unlike more traditional adult tumours, which are largely driven by genomic mutations (Khan and Helman, 2016), epigenetically driven childhood cancers have proven resistant to classical therapeutic target identification approaches, directed against mutations in oncogenes (Johnsen et al., 2018). Furthermore, existing therapies for neuroblastoma have severe and sometimes long-term side effects that include an increased risk of second malignancies (Applebaum et al., 2017).

Neuroblastoma begins *in utero* and the disease is predominantly diagnosed in the first year of life. Despite being rare, it accounts for 8–10% of all diagnosed childhood cancers (Stack et al., 2007; Dreidax et al., 2014; Henrich et al., 2016). However, due to its aggressiveness, neuroblastoma is responsible for 14% of all childhood cancer deaths (Stack et al., 2007). Neuroblastoma is divided into risk groups based on criteria which include: the age of the patient at diagnosis, International Neuroblastoma Risk Group (INRG) tumour stage and MYCN copy number status (Cohn et al., 2009; Monclair et al., 2009). Patients with low-risk (stage 1, 2 and 4s) and intermediate (stage 3) neuroblastoma have event free survival (EFS) of up to 90%, contrary to high-risk patients with EFS of less than 50% (Smith and Foster, 2018; Meany, 2019). Amplification of the MYCN oncogene, which occurs in approximately 20% of cases (Huang and Weiss, 2013), is one of the clearest markers for identifying high-risk neuroblastoma patients, regardless of disease stage. MYCN amplification results in increased cellular proliferation and growth, decreased apoptosis, poor differentiation, and increased vascularity of the tumours (Gustafson and Weiss, 2010). Additionally, is also associated with advanced stage disease, an overall poor prognosis, and therapy resistance (Shimada et al., 2001), with MYCN-amplified tumours being resistant to current therapeutic approaches. Multiple studies have shown that MYCN exerts its functions through interactions with the epigenetic machinery (Domingo-Fernandez et al., 2013; He et al., 2013; Puissant et al., 2013; Carter et al., 2015; Duffy et al., 2015; Yang et al., 2015; Duffy et al., 2016; Henrich et al., 2016; Duffy et al., 2017; Felgenhauer et al., 2018). Therefore, deeper understanding of MYCN dependent epigenetic vulnerabilities provides a novel route for targeted therapies in neuroblastoma.

We investigated the MYCN-related epigenetic signalling network, and the potential of epigenetic lead compounds as therapeutic agents for the treatment of high-risk MYCN amplified neuroblastoma. MYCN is a transcription factor that binds to the promoters of genes critically involved in neuroblastoma oncogenesis (Duffy et al., 2015). We performed an unbiased genome-wide MYCN target gene screen using chromatin immunoprecipitation sequencing (ChIP-seq) to identify MYCN-epigenetic cross-talk, and combined it with a phenotypic screen of small molecule epigenetic targeting compounds. These approaches converged on a number of promising hit compounds which were further characterised.

## Results

### ChIP-seq Identifies Epigenetic Regulators as MYCN Targets

**FIGURE 1:**
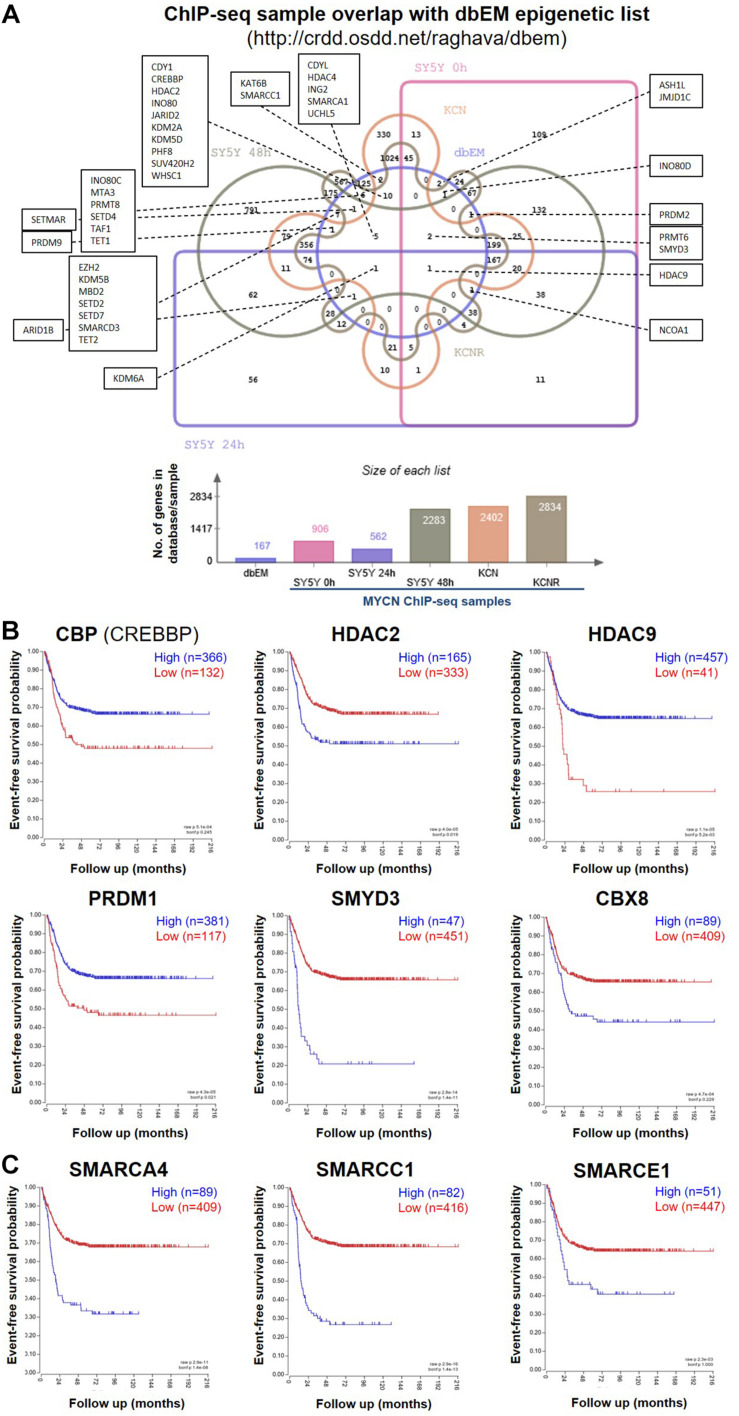
MYCN ChIP-seq epigenetic hits and their survival curves. **(A)** Venn diagram showing the overlap of genes called as genomic MYCN targets (ChIP-seq) in SY5Y-MYCN (un-induced Dox-, 24 h Dox+ and 48 h Dox+), KCN and KCNR cell lines and known epigenetic genes (Singh Nanda et al., 2016) (dbEM, Database of Epigenetic Modifiers, http://crdd.osdd.net/raghava/dbem). The Venn diagram was generated using jvenn (Bardou et al., 2014) (http://jvenn.toulouse.inra.fr/app/index.html). **(B)** Kaplan-Meier survival curves showing the predictive strength of the expression levels of the CBP, HDAC2, HDAC9, PRDM1, SMYD3, and CBX8 mRNAs in neuroblastoma tumours on patient outcome. **(C)** Kaplan-Meier survival curves showing the predictive strength of the expression levels of SMARC genes in neuroblastoma tumours on patient outcome. The SMARCs shown were predicted to be upstream regulators of MYCN’s genomic targets (ChIP-seq). SMARCC1 was also a direct MYCN target (ChIP-seq). All survival curves were generated using the SEQC (Zhang et al., 2015) 498 neuroblastoma tumour dataset in the R2: Genomics Analysis and Visualization Platform (http://r2.amc.nl).

To identify MYCN’s epigenetic-related genomic targets, we mined our MYCN ChIP-seq datasets (Duffy et al., 2015) for known epigenetic genes (ArrayExpress, www.ebi.ac.uk/arrayexpress, accession number E-MTAB-4100). These datasets comprise MYCN ChIP-seq data from the patient matched MYCN amplified cell lines KCN (from a primary tumour at diagnosis) and KCNR (from a secondary tumour after relapse), and of a time-course of MYCN overexpression in the MYCN inducible cell line SY5Y-MYCN at the following time-points: Un-induced (0h), 24h and 48h. The MYCN overexpression achieved in this system is similar to overexpression caused by MYCN gene amplification. To identify the epigenetic-related genes bound by MYCN, we intersected the MYCN ChIP-seq targets with the 167 gene dbEM, Database of Epigenetic Modifiers (Singh Nanda et al., 2016)^1^. Forty-two of the dbEM genes (25%) were identified in our MYCN ChIP-seq datasets (Figure 1A and Table 1), confirming that MYCN protein targets a relatively high proportion of epigenetic-related genes.

**TABLE 1:** Comparison of ChIP-seq MYCN target gene dataset with a curated epigenetic regulators lists.

MYCN ChIP-seq hits overlapped with the dbEM database of epigenetic modifiers				
CREBBP (CBP)	HDAC9	SUV420H2	JMJD1C	EZH2
HDAC2	HDAC4	SETD2	KDM5B	MBD2
WHSC1	PRMT8	SETD7	CDYL	PRDM2
SMARCA1	SETD4	SETMAR	INO80C	PRDM9
UCHL5	TAF1	MTA3	NCOA1	JARID2
SMARCD3	TET1	ING2	KDM2A	ARID1B
SMARCC1	CDY1	INO80	KDM5D	
ASH1L	PHF8	PRMT6	KDM6A	
TET2	KAT6B	SMYD3	INO80D	
**42 / 167 genes**				

*Forty-two epigenetic-related genes which were MYCN genomic targets. Gene list obtained by overlapping the 167 gene dbEM, Database of Epigenetic Modifiers (http://crdd.osdd.net/raghava/dbem) with our MYCN ChIP-seq hits (Figure 1A; Duffy et al., 2015, 2016; Singh Nanda et al., 2016).*

To identify epigenetic proteins which formed protein-protein interactions with MYCN we also mined our MYCN interactome data in SY5Y-MYCN cells (Duffy et al., 2015; Duffy et al., 2017). Thirty-four epigenetic proteins physically, directly or as a part of a complex, bound to MYCN protein (Supplementary Table 1), including three epigenetic regulators whose coding genes were genomic targets of MYCN (ChIP-seq); CBP (official gene symbol CREBBP), HDAC2 and CBX8. Expression of these genes was assessed for survival correlations in the SEQC dataset (Zhang et al., 2015) of 498 neuroblastoma patients, using the R2: Genomics Analysis and Visualization Platform^2^. All six MYCN target genes (ChIP-seq) which were common to at least two of the three lists (Supplementary Table 1), had prognostic value for neuroblastoma patient outcome, when patients were segregated according to expression of these genes (Figure 1B).

### Analysis of the Other Known Transcriptional Regulators of MYCN’s Genomic Targets Reveals Epigenetic Regulation

To further address the significance of epigenetic mechanisms and their potential interplay with MYCN regulatory networks, we next examined MYCN’s genomic targets (ChIP-seq) for known epigenetic co-regulators, using Ingenuity pathway analysis (IPA). IPA has a manually curated database of transcriptional regulators (Krämer et al., 2014), and certain epigenetic proteins were consistently recognised as inferred upstream regulators of MYCN’s target genes in all ChIP-seq datasets (Table 2). The common inferred transcriptional regulators (ITRs) were enriched for key regulators of chromatin structure (Table 2), including HDAC and SMARC genes. It has previously been shown that expression levels of 11 HDAC members in primary neuroblastomas are correlated with NB prognosis and stage (Oehme et al., 2009; Figure 1B) and that HDAC2 functionally interacts with MYCN (Lodrini et al., 2013; Duffy et al., 2016). The SMARC gene family regulates transcription by altering the chromatin structure around certain genes. This family of proteins still remains poorly understood, although it has been shown that mutations that inactivate their subunits are found in nearly 20% of human cancers (Hohmann and Vakoc, 2014). All six of the SMARC genes, identified as being ITRs of MYCN’s genomic targets (ChIP-seq), were able to segregate neuroblastoma patients by outcome, according to the level of SMARC gene expression in tumours (Figure 1C and Supplementary Figure 1A). For four of these SMARC genes (SMARCA4, SMARCC1, SMARCE1 and SMARCB1) high expression was associated with poor outcome.

**TABLE 2:** Inferred Transcriptional Regulators (ITRs) for MYCN ChIP-seq samples belong to HDAC and SMARC families of epigenetic regulators.

Inferred Transcriptional Regulator (ITR)	Dataset	*p*-value of overlap
HDAC family	KCNR	2.04E–07
	24h SY5Y-MYCN	5.63E–06
	48h SY5Y-MYCN	5.68E–06
	KCN	1.61E–05
HDAC1	KCNR	3.20E–03
	KCN	6.02E–03
HDAC2	KCN	4.06E–05
	KCNR	4.10E–05
	48h SY5Y-MYCN	1.83E–02
	0h SY5Y-MYCN	4.51E–02
HDAC4	48h	1.20E–07
	KCN	6.20E–05
	KCNR	1.30E–04
	0h SY5Y-MYCN	1.18E–03
	24h SY5Y-MYCN	2.70E–03
HDAC5	48h	2.98E–02
HDAC6	0h SY5Y-MYCN	1.36E–02
SMARCA1	KCN	4.04E–02
SMARCA4	0h SY5Y-MYCN	2.49E–02
	KCNR	2.85E–02
SMARCB1	KCN	1.60E–02
SMARCC1	0h SY5Y-MYCN	4.96E–03
	KCN	3.07E–02
	48h SY5Y-MYCN	4.56E–02
SMARCD3	24h SY5Y-MYCN	3.83E–04
	0h SY5Y-MYCN	9.48E–04
	48h SY5Y-MYCN	1.61E–02
	KCNR	2.65E–02
SMARCE1	0h SY5Y-MYCN	6.08E–03
CREB1 (epigenetic related)	0h SY5Y-MYCN 24h SY5Y-MYCN 48h SY5Y-MYCN KCN KCNR	4.46E–08 1.28E–07 2.99E-16 1.56E–08 1.90E–08

*MYCN ChIP-seq datasets are from MYCN-amplified KCN and KCNR cell lines and a MYCN overexpression time-course (un-induced [0 h], 24 h, and 48 h induction) in SY5Y-MYCN cells.*

### Screening an Epigenetic Library in MYCN Amplified Cells Reveals a Broad Vulnerability to Epigenetic Compounds

Having identified novel MYCN-epigenetic links and confirmed that MYCN can interact with epigenetic genes, we next sought to identify small molecules directed against epigenetic regulators with therapeutic potential for treatment of MYCN amplified neuroblastoma. To achieve this, in a non-biased manner, we employed a curated compound library generated by the Structural Genomics Consortium (SGC)^3^, which is comprised of 45 epigenetic targeting compounds (Supplementary Table 2). The compound library was screened on the IMR32 cell line, and resulting changes to cell phenotype and viability were assessed. IMR32 cells harbour a high level of MYCN amplification with a consequent increase in MYCN expression levels (Tumilowicz et al., 1970; Duffy et al., 2014, 2015) (Supplementary Figure 1B). Conversely, c-MYC levels are almost undetectable in IMR32 cells (Duffy et al., 2014, 2015; Supplementary Figure 1B), qualifying this cell line as a model where MYCN-dependent epigenetic regulatory networks are not obscured by interplay with often overlapping c-MYC-dependent regulatory networks.

**FIGURE 2:**
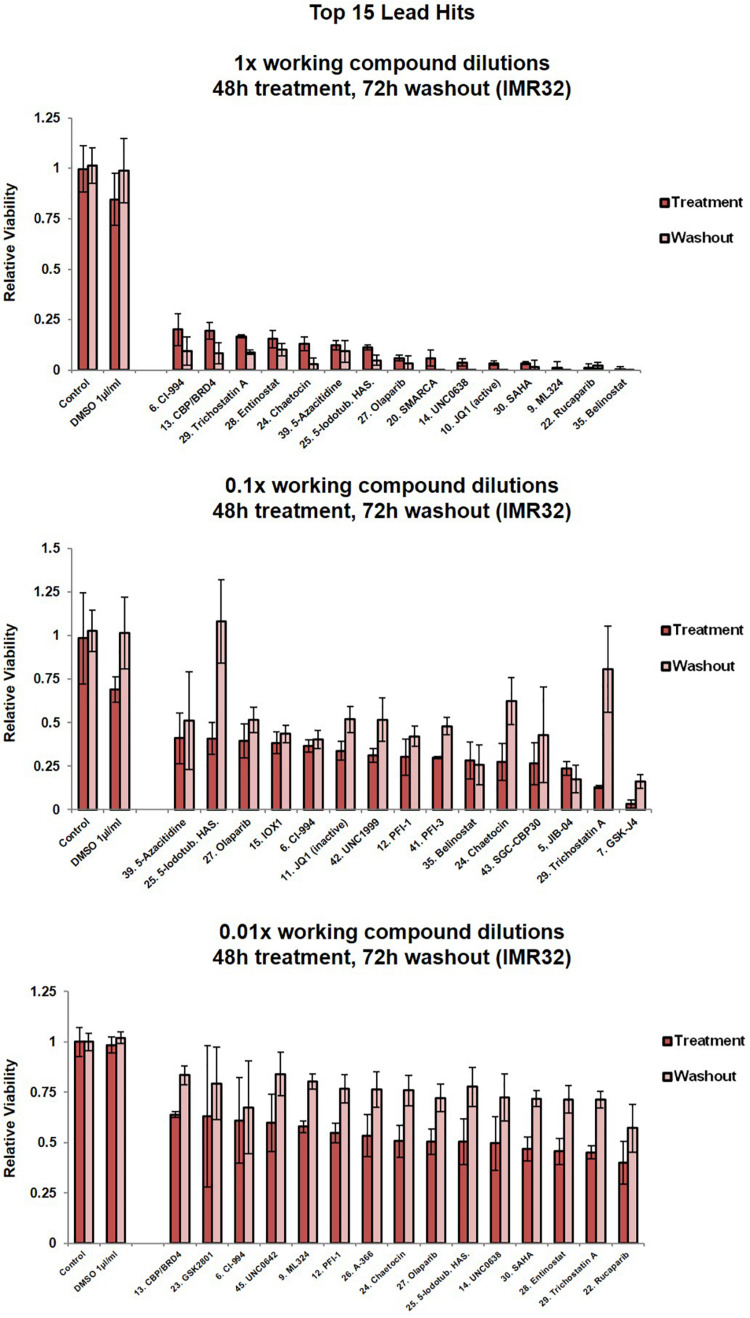
Cell viability assessment for IMR32 cells upon treatment with the top 15 lead SGC library compounds. Compound dilutions are indicated above histograms (1x, 0.1x or 0.01x). Treatment bars – viability upon 48h treatment. Washout bars – viability 72 h upon washout, relative to corresponding untreated control at the same time point. For results from all 45 compounds in the library please see Supplementary Figure 1C. Error bars indicate the mean ± SD.

We analysed the phenotypic response of IMR32 cells to treatments with library compounds at concentrations that showed physiological/cellular activity in other *in vitro* model systems. Since properties of the leads greatly varied (molecular weight, solubility, IC_50_), treatments were performed in serial dilutions (1x, 0.1x and 0.01x) of the library working stock concentrations (Supplementary Table 2), corresponding to concentration ranges from 0.05 mM for potent histone methyltransferase inhibitors JIB-04 (Wang et al., 2013) and chaetocin (Cherblanc et al., 2013) to 1M for the weak aliphatic HDAC inhibitor valproic acid (Göttlicher et al., 2001; Dokmanovic et al., 2007). The 1x dilution is the suggested SGC working concentration for each compound. Cell morphology was assessed after 48 h (Supplementary Figure 2B). The 1x compound dilutions resulted in dramatic changes of cell morphology (Supplementary Figure 2B) or almost complete wipe-out of cell numbers in many cases (Figure 2, Supplementary Figure 1C), except the DNA-methyltransferase inhibitor, Decitabine. However, Decitabine did produce similar cellular responses to the other compounds upon prolonged 72 hour treatment, namely cell debris, reduced cell surface, lost axonal protrusions and clustered cells with highly heterochromatic nuclei (Supplementary Figure 2C). Interestingly, treatments with JQ-1 (BET-bromodomain inhibitor) and LLY-507 (inhibitor of SMYD2 protein lysine methyltransferase activity) led to a reduction of contact inhibition and clustering of the cells. SMYD2 expression in neuroblastoma tumours was also predictive of patient outcome (Supplementary Figure 2D).

Five compounds reduced IMR32 relative cell viability by at least 50% at nanomolar concentrations (0.01x dilution), i.e., UNC0638 (10 nm), SAHA (10 nm), entinostat (5 nm), TSA (5 nM) and rucaparib (100 nM) (Figure 2). These compounds are characterised as a H3K9me2 methyltransferase inhibitor (Vedadi et al., 2011), pan-HDAC inhibitor (Marks, 2007), HDAC1/HDA3 inhibitor (Saletta et al., 2014), pan-HDAC inhibitor (Hřebačková et al., 2009) and poly-ADP-ribosylation inhibitor of histone H1 (D’Amours et al., 1999), respectively. Interestingly, these potent compounds fall across the major epigenetic regulation groups (writers, readers and erasers), again implying a complex interplay of epigenetic regulatory mechanisms with neuroblastoma biology.

A number of the identified MYCN-related epigenetic hits (Figures 1A–C and Tables 1, 2 and Supplementary Table 1) were targets of compounds within the library and where this was the case, compound treatment induced a pronounced loss of cell viability (Figure 2, Supplementary Figure 1C). Specifically, SMARCA (compound 20), CBP (compounds 13, 40 and 43), SMYD (compound 44) and HDACs (compounds 6, 18, 28, 29, 30, 35, 36, and 37). This observation suggests that epigenetic therapeutics could be used to target MYCN oncogenic networks.

Since epigenetic compounds can generate long term-effects based on modulating epigenetic marks that may persist beyond the treatment period, we employed a washout strategy to assess whether the cells were able to recuperate after each treatment, or if the compounds had sustained effects. Alamar Blue viability assays are non-toxic enabling multi-point monitoring (Rampersad, 2012). After 48 h compound treatments, media containing the drug was removed and the cells were allowed to grow in standard media for a further 72 h. Crucially, even after the additional 72 h washout period, and in the 1x dilution condition the vast majority of compounds resulted in cell viability remaining at or below the post-48h-treatment level (Figure 2, Supplementary Figure 1C). The epigenetic compounds produced long lasting effects, with cells not able to recommence proliferation. For almost half of the compounds (1x dilution condition), a further decline in the cell viability could be observed after the washout period (Figure 2, Supplementary Figure 1C).

### Selective CBP/p300 Inhibitors Strongly Reduce the Viability of MYCN-Amplified Cells

**FIGURE 3:**
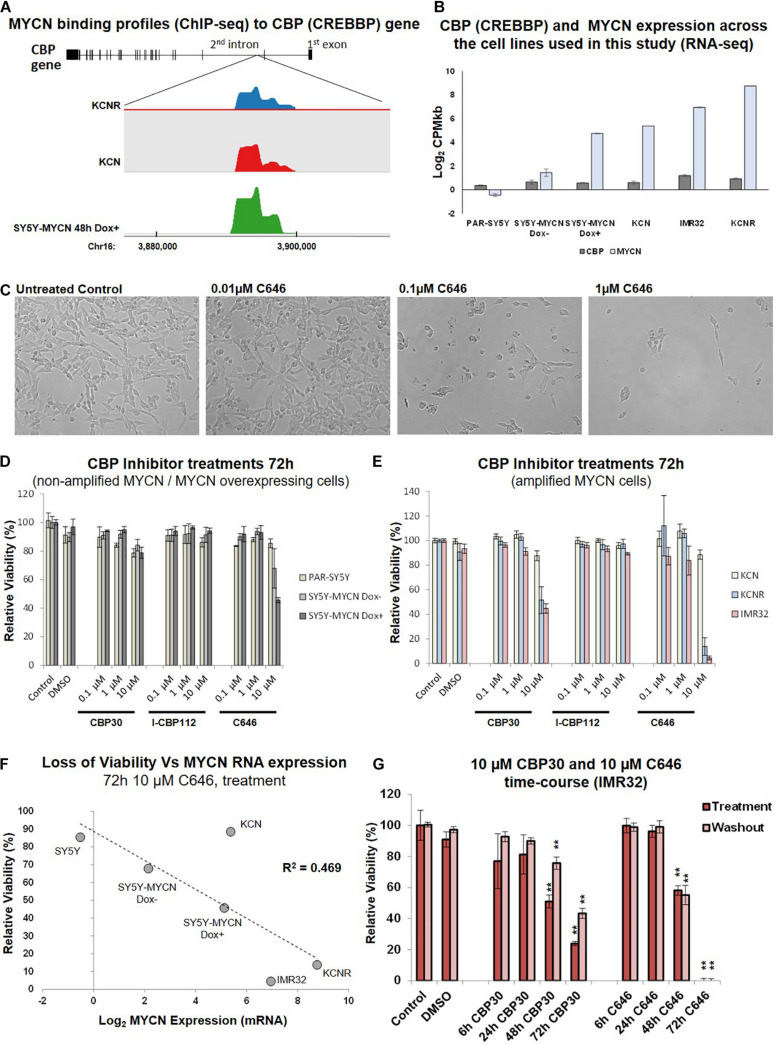
CBP inhibition across a neuroblastoma cell line panel. **(A)** MYCN binding enrichment within the second intron of the CBP gene. Top – CBP gene schematic representation. Middle – Probe enrichment visualisation for KCNR, KCN, and SY5Y-MYCN 48 h Dox induced representative datasets using the SeqMonk tool. Bottom -genomic coordinates. **(B)** CBP and MYCN transcript expression levels across the cell line panel. Read counts per million adjusted by gene length in kilobases (CPMkb), obtained from our previously published RNA-seq of these cell lines (Duffy et al., 2015, 2017). **(C)** Images of IMR32 cells after 48 h treatment with C646. Images were taken with 40x magnification. **(D)** Relative viability (Alamar Blue assay) upon CBP inhibitor treatment (CBP30, I-CBP112 and C646) of MYCN single copy cell lines, including doxycycline inducible MYCN cell line SY5Y-MYCN. **(E)** Relative viability (Alamar Blue assay) upon CBP inhibitor treatment (CBP30, I-CBP112 and C646) of MYCN amplified cell lines, including doxycycline inducible MYCN cell line SY5Y-MYCN. **(F)** Correlation of relative viability loss upon C646 treatment (Alamar Blue assay) and MYCN mRNA expression (Log_2_ fold change) obtained from RNA-seq (Duffy et al., 2015). *R*^2^-value reported on the panel is from a Pearson Correlation Coefficient analysis. **(G)** Relative viability (Alamar Blue assay) of C646 time-course (6 h, 24 h, 48 h, and 72 h) treatment of MYCN amplified IMR32 cells. Viability results immediately after treatment (Treatment) and after an additional 48 h in the absence of C646 after treatment ended (Washout) are shown. Paired sample t-test values are designated with asterisks above bars. *P* ≤ 0.05 - *; *P* ≤ 0.01 - **. Error bars indicate the mean ± SD.

From the library’s lead compounds, we further evaluated CBP/p300 bromodomain inhibitors as potential MYCN amplified neuroblastoma therapeutics, since CBP (CREBBP) was prominent in our MYCN-related -omic and neuroblastoma patient outcome analysis. CBP was identified as a MYCN target gene in all MYCN ChIP-seq datasets (Figure 3A and Table 1, Supplementary Table 1), and CBP was a protein-protein interactor of MYCN (Duffy et al., 2015, 2016) in SY5Y-MYCN cells (Supplementary Table 1, Supplementary Figure 3A). CBP’s co-factor p300 (EP300) was also identified as being MYCN bound (Supplementary Table 1 and Supplementary Figure 3A). Similarly, CREB1, which acts in concert with CBP, is also a member of MYCN’s transcriptional regulatory network, as revealed by IPA’s ITR analysis of the ChIP-seq datasets (Table 2). In line with this observation on CBP, we have previously shown that inhibiting β-catenin binding to CBP in neuroblastoma cells alters their proliferative potential (Duffy et al., 2016).

CBP mRNA was consistently expressed in the cell lines utilised in this study (Duffy et al., 2015; Figure 3B), and more broadly across a panel of 39 neuroblastoma cell lines (Harenza et al., 2017) (Supplementary Figure 2A). Conversely, MYCN expression levels varied across the cell lines selected for this study (Duffy et al., 2014, 2015, 2016, 2017; Schwarzl et al., 2015; Figure 3B). CBP, CREB1 and p300 expression were individually prognostic for neuroblastoma patient outcome, with patients with low CBP, CREB1 or p300 mRNA expression only having an event-free survival rate of approximately 50% (Figure 1B and Supplementary Figure 3B). The library screen revealed that IMR32 cell viability is reduced upon CBP inhibition (Figures 2, 3C).

Given the well-established link between the MYCN oncogene and neuroblastoma patient outcome, the response of neuroblastoma cell lines with varying MYCN status to CBP/p300 inhibition was assessed. The effects of three inhibitors: C646, CBP30 and I-CBP112 (Figure 2, Supplementary Figure 1C, epigenetic library compound 33, 40 and 43) (Bowers et al., 2010; Hay et al., 2014; SGC, 2020), on relative cell viability were assessed in a neuroblastoma cell line panel. The cell line panel covers the full spectrum of neuroblastoma’s MYCN genetic backgrounds, ranging from non-amplified to MYCN amplified cells and from almost undetectable expression of MYCN to highly expressing cells (Duffy et al., 2014, 2015). While I-CBP112 did not exert a strong effect, both C646 and CBP30 reduced cell viability, with the cells being most susceptible to C646 treatment (Figures 3D,E). Interestingly, non-amplified MYCN cells (PAR-SY5Y, Dox- and Dox+ SY5Y-MYCN) and the MYCN amplified line with the lowest MYCN expression KCN were almost resistant to CBP30 treatment (Figures 3D,E). Conversely, the cell lines with high MYCN amplification IMR32 and KCNR responded strongly to 10μM CBP30 with significant reductions of cell viability, 55% and 49% loss of viability, respectively (Figure 3E). This pattern was repeated with 10 μM C646, with non-amplified MYCN cell lines showing minimal loss of viability, while MYCN amplified cell lines IMR32 and KCNR had a loss of viability of 95% and 87% respectively.

Responsiveness to 10 μM C646 was MYCN dose-dependent (Figure 3F), with the level of MYCN expression across the cell lines positively correlating to loss of viability (Pearson Correlation Coefficient, *R*^2^ = 0.469). While parental (PAR-)SY5Y and un-induced (Dox^–^) SY5Y-MYCN cells were practically resistant to 10 μM C646, SY5Y-MYCN cells induced to overexpress MYCN (Dox^+^) had cell viability reduced to 45% (Figures 3D–F). Within the same genetic background, elevated MYCN expression significantly sensitises the cells to C646 (10 μM C646, Dox+ versus Dox- SY5Y-MYCN, *t*-test, *p*-value = 0.0190; Figure 3D) and even a modest increase in MYCN expression (Figure 3B) led to increased C646 sensitivity (10 μM C646, parental SY5Y versus Dox- SY5Y-MYCN, *t*-test, *p*-value = 0.0470; Figure 3D).

As was the case for CBP30, the KCN cell line was only very weakly reactive to C646 treatments (88% relative viability for 10 μM C646). Although KCN cells are MYCN amplified, the extent of amplification (and MYCN expression) is much lower than in IMR32 and KCNR cells (Duffy et al., 2015). KCN cells are derived from the primary tumour of a 1 month old infant, representing a less aggressive form of neuroblastoma than the metastatic tumour derived KCNR and IMR32 cells. KCNR is a patient matched cell line to KCN, being derived after relapse from a secondary tumour from the same individual (one year later). Therefore, the differential response to CBP/p300 inhibition between KCN and KCNR are not due to the initial mutational/epigenetic spectrum that gave rise to neuroblastoma in this patient, but likely from subsequent alterations which occurred between the primary and secondary tumours. One such characterised alteration is an over ten-fold increase in MYCN mRNA expression (Duffy et al., 2015; Figure 3F). Due to the lack of cytotoxic effects in the analysed neuroblastoma cell panel, compound I-CBP112 was excluded from further characterisation, while CBP30 and C646 were characterised in more detail. CBP/p300 inhibition shows potential as a therapeutic approach for advanced drug resistant MYCN amplified metastatic neuroblastoma tumours.

### Temporal and IC_50_ Characterisation of CBP30 and C646 in IMR32 Cells

We next determined the temporal profile of the activity for 10 μM CBP30 and C646 by treating IMR32 cells from 6 h to 72 h (Figure 3G). We observed no significant effect for both leads during short treatments (6 h and 24 h), while prolonged treatments (48 h and 72 h) had a significant and pronounced effect on cell viability (Figure 3G). C646 exerted switch-like temporal behaviour with a difference in relative viability between 48 h and 72 h treatments. Washout (48 h) experiments did not reveal any significant change in the cell viability when compared to their corresponding treatment, showing that cells are unable to recover from treatment even after the removal of the inhibitor from the growth media (Figure 3G).

**FIGURE 4:**
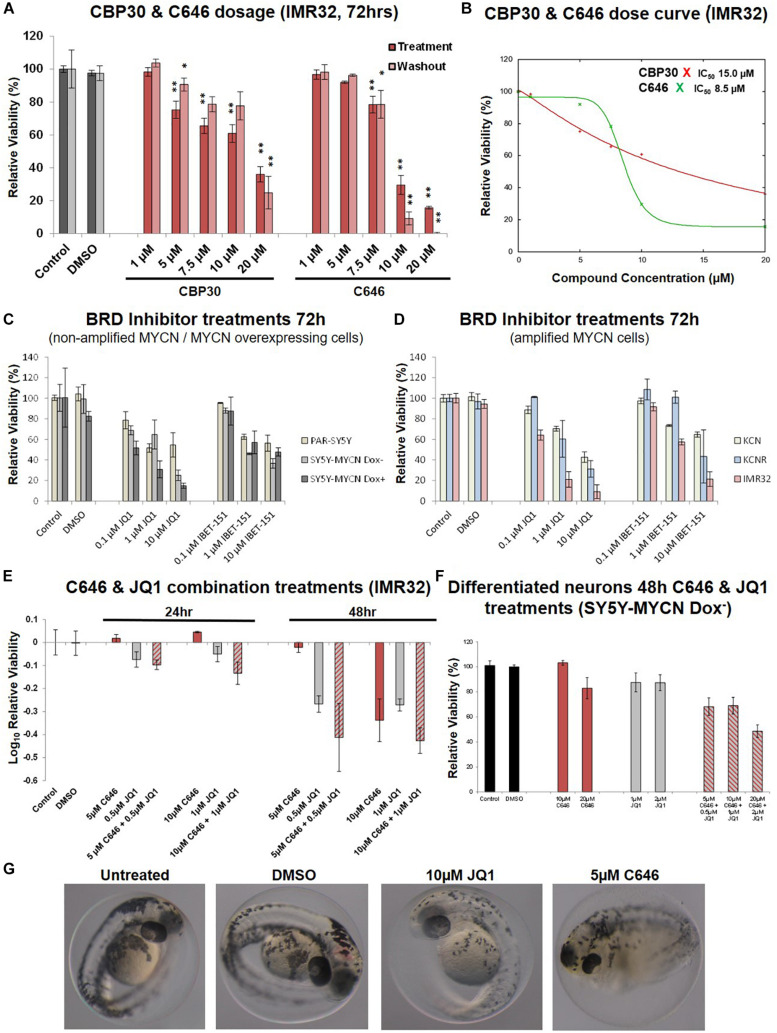
Dose curves, combination treatments, neuronal and phenotypic screens. **(A)** Expanded dose response assessment of the inhibitors C646 and CBP30 in MYCN amplified IMR32 cells. Relative cell viability was determined using Alamar Blue assays. Viability results immediately after the 72 h treatment (Treatment) and after an additional 48 h in the absence of inhibitors after treatment ended (Washout) are shown. Paired sample *t*-test values are designated with asterisks above bars. *P* ≤ 0.05 - *; *P* ≤ 0.01 - **. Error bars indicate the mean ± SD. **(B)** IC_50_ estimate for CBP30 and C646 for the 48h treatment of IMR32 cells. Asterisks correspond to X axis drug concentrations (μM) and Y axis relative cell viability (%). Curve fitting performed with IC Toolkit (http://ic50.tk/index.html). **(C)** Relative viability (Alamar Blue assay) upon BRD4 inhibitor treatment (JQ1 and I-BET151) of MYCN single copy cell lines, including doxycycline inducible MYCN cell line SY5Y-MYCN. **(D)** Relative viability (Alamar Blue assay) upon BRD4 inhibitor treatment (JQ1 and I-BET151) of MYCN amplified cell lines, including doxycycline inducible MYCN cell line SY5Y-MYCN. **(E)** Relative viability (Alamar Blue assay) upon combination CBP and BRD4 inhibitor treatment (C646 and JQ1) of MYCN amplified IMR32 cells. **(F)** Single agent and combination treatment (C646 and JQ1) of differentiated neurons (pre-inhibitor treatment, 11 day RA treated un-induced SY5Y-MYCN cells). Log_10_ relative viability (Alamar Blue assay) upon 48 h treatments combination CBP and BRD4 inhibitor treatment (C646 and JQ1) of MYCN amplified IMR32 cells. Error bars indicate the mean ± SD. **(G)** Zebrafish embryos treated for 24 h (28 h post fertilisation [hpf]- 52hpf) with epigenetic targeting compounds (JQ1 and C646). Magnification is 10x, images from 52 hpf. Melanocytes appear black due to their endogenous melanin. Otic vesicles (large black circles) mark the anterior pole (head) of the embryos.

An expanded dose-dependent response analysis was performed by treating IMR32 cells with 1 – 20 μM CBP30 or C646 for 72 h (Figure 4A). Washout relative cell viability was also determined 48 h after the 72 h treatment (Figure 4A). For both leads, concentrations above 5 μM were required to induce major cytotoxic effect in IMR32 cells (Figure 4A). Even after washout the reduction in viability in the high-dose treatments was maintained with the reduction continuing to intensify even in the absence of the inhibitors (Figure 4A). The response curves and IC_50_ values of each compound were estimated using the IC50 Toolkit^4^. The estimated IC_50_ values for C646 and CBP30 were 8.5 μM and 15 μM, respectively (Figure 4B), confirming that C646 is the more potent lead. C646 had a sigmoid dose response curve, contrary to CBP30’s linear dose response curve (Figure 4B). Viability responses to C646 exhibited switch-like behaviour, both in terms of its temporal response and dose response profiles.

### CBP and BRD4 Inhibitor Combination Treatments

We next assessed whether targeting multiple epigenetic vulnerabilities simultaneously would have additive or synergistic effects on neuroblastoma cell viability. The compound library results showed that BET-bromodomain BRD4 inhibition strongly reduced IMR32 cell viability (compounds 10, 21, 34 and 41, Figure 2, Supplementary Figure 1C). Furthermore, a compound [CBP/BRD4 (0383)] co-targeting BRD4 (reader) and the related CBP protein (writer) also resulted in a strong loss of cell viability (compound 13, Figure 2). Like CBP and its CREB and p300 co-factors, BRD4 expression level is predictive of neuroblastoma patient outcome (Figure 1B and Supplementary Figure 3B). We therefore tested BRD4 and CBP combination treatment in IMR32 cells. First, to confirm the effects of BRD4 inhibition in neuroblastoma cells, a dose course of the BRD4 inhibitors JQ1 and I-BET were assessed in the cell line panel (Figures 4C,D). Mirroring the library screening (Figure 2, Supplementary Figure 1C) both inhibitors strongly reduced IMR32 cell viability, with JQ1 being the more potent (Figure 4D). Similar to CBP inhibition, BRD4 inhibition tended to reduce viability to a greater degree in high MYCN amplified cells (KCNR and IMR32). Although, non-amplified MYCN cells, MYCN overexpressing cells (SY5Y-MYCN Dox+) and early stage primary MCYN cells (KCN) were more responsive to JQ1 inhibition than they were to CBP inhibition (Figures 3C,D, 4C,D).

Having confirmed the BRD4 inhibitors effect across the cell line panel, we next evaluated potential synergy between these compounds. IMR32 cells were treated with single agents at their near IC_50_ concentrations, C646 (10 μM and 5 μM) and JQ1 (1 μM and 0.5 μM), as well as with their combinations. Combining JQ1 and C646 resulted in slight additive effects at 24 h and 48 h (Figures 4E,F), although at 48hr this was non-significant, *p*-value = 0.1301 (paired sample *t*-test). Combination treatments were also assessed in the MYCN non-amplified cell line SY5Y-MYCN with Dox inducible MYCN expression. Once again combination treatment only produced a mild additive effect (Supplementary Figure 3C).

### Zebrafish Developmental Screens and Lack of Cytotoxicity of C646 in Differentiated Neurons

To further asses the lead compounds, we examined how well tolerated they were in zebrafish developmental phenotypic screens. Due to its dynamic nature, embryonic development tends to be more sensitive to pharmaceutical perturbations than non-developmental stages, with responses in rapidly dividing embryonic cells often recapitulating those observed in rapidly proliferating cancer cells. Twenty-four hour treatments with IBET-151, CBP30 and I-CBP112 (concentrations 1, 5, and 10 μM) were well tolerated and resulted in no obvious phenotype changes (Supplementary Figure 4A). While 1 μM C646 was well tolerated, 5 μM C646 treated embryos had an overall distorted appearance and an observable increase in heart rate (Figure 4G). Furthermore, there was 100% lethality in embryos treated with 10 μM C646. While there were no obvious gross phenotypic effects of 1 μM and 5 μM JQ1 treatment, 10 μM JQ1 resulted in a reduced chromatophores population by 52 h post fertilisation (hpf), resulting in significant embryo depigmentation (Figure 4G). Like neuroblastoma precursor cells (neuroblasts), melanocytes (chromatophore cell type) also originate from the transient embryonal neural crest tissue (Kuriyama and Mayor, 2008). Interestingly, inhibition of Wnt signalling similarly affected chromatophore (melanocyte) development in a similar screen (Duffy et al., 2016). While embryonic development is known to be more sensitive to perturbation than post-developmental stages, the potency of C646 and JQ1 should be carefully considered when assessing their utility as therapeutics for childhood cancers.

Cancer therapy, including chemotherapeutic agents, may affect the nervous system in a deleterious manner. In patients this can lead to numerous sequelae, such as chemotherapy-related encephalopathy, meningitis, chronic pain syndrome, psychiatric disorders, neuropathy (Chamberlain, 2010). Often, these neurological problems are associated with neurotoxicity. Laboratory differentiated neuroblastoma cell lines have been used extensively to screen novel compounds for neurotoxic properties and associated mechanisms. Importantly, the response of neuroblastoma cells to compounds and drugs exposure may differ from that of neurons. The differentiation potential of neuroblastoma cell lines into mature neurons, enables the pharmacological and functional differences between neurons and their blast cell counterparts to be assessed (LePage et al., 2005; Duffy et al., 2016, 2017).

To assess the likelihood of unintended neurotoxicity from the clinical use of C646 and JQ1, we analysed the effect on viability of long-term differentiated SY5Y-MYCN cells upon treatment with these inhibitors as single agents or in combinations. Ahead of the epigenetic drug treatment, SY5Y-MYCN cells were treated with 1 μM all-trans retinoic acid (RA) for 11 days. The differentiation capacity of RA has been well established, with extensive outgrowth of neurites and expression of neuron-specific markers (Encinas et al., 2000; Cheung et al., 2009; Duffy et al., 2016, 2017), and we have previously shown that the SY5Y-MYCN cell line also undergoes neuronal differentiation in response to RA (Duffy et al., 2014, 2017). Differentiated SY5Y-MYCN neurons were fully resistant to 10 μM C646, and only weakly responsive to 20 μM C646 concentration (Figure 4F). Similarly, the differentiated neurons were more resistant to 1 μM JQ1 treatment (Figure 4F) than their undifferentiated counterparts (Supplementary Figure 3C). Forty-eight hour 10 μM C646 and 1 μM JQ1 combination treatment reduced cell viability to approximately 25% in both Dox^–^ and Dox^+^ SY5Y-MYCN cells (Supplementary Figure 3C), whereas differentiated neurons were more tolerant of the combination treatment with approximately 70% remaining viable (Figure 4F). Taken together, these results demonstrate that the C646 and JQ1 epigenetic targeting compounds are better tolerated by differentiated neurons than their more stem-like neuroblastoma cell counterparts. Furthermore, C646 is better tolerated by neurons than JQ1, with 10 μM C646 having no effect on neuronal cell viability.

## Discussion

Epigenetic alterations are strongly associated with the development of cancer, and compounds targeting epigenetic modifier proteins are increasingly being assessed as anti-cancer therapeutic agents (Juergens et al., 2011). Integration of multi-omic MYCN datasets revealed strong support for the hypothesis that targeting epigenetic regulatory mechanisms in MYCN amplified neuroblastoma will likely provide beneficial future therapeutic strategies. Functional support for the applicability of epigenetic targeting compounds was provided by the fact that 43 of the 45 epigenetic library compounds reduced the viability of MYCN amplified neuroblastoma cells. The high responsiveness rate (96%) across a range of epigenetic modulators, confirms a prominent role for epigenetic alterations as drivers of neuroblastoma oncogenesis and progression. Viability assays revealed that modulation of epigenetic regulatory mechanism takes time to exert its effect, but at 1x concentration given sufficient time, dramatic reductions in NB cell viability can be achieved.

In addition to signalling previously reported cross-talk between MYCN and HDAC (Lodrini et al., 2013; Duffy et al., 2016; Fabian et al., 2016; Phimmachanh et al., 2020), we revealed novel MYCN-epigenetic interactions, including those with SMARC genes. SMARC genes were identified as inferred transcriptional regulators of MYCN-bound genes (ChIP-seq), their expression showed strong correlation to neuroblastoma outcome, and SMARCA inhibition strongly reduced IMR32 cell viability.

The SMARC gene family comprises a family of proteins, that display both helicase and ATPase activities, and which play roles in regulation of transcription of certain genes by altering the chromatin structure around those genes (Zofall et al., 2006). Mutations that inactivate SMARC subunits are found in nearly 20% of human cancers, driving aberrant growth (Hohmann and Vakoc, 2014). Certain acute leukaemias and small cell lung cancers, which lack SMARC mutations (similar to neuroblastoma) can be vulnerable to inhibition of SMARCA4 (Hohmann and Vakoc, 2014).

MYCN amplified neuroblastoma cells are resistant to retinoid-based differentiation therapy (Duffy et al., 2017). Interestingly, cell lines lacking SMARCA4 from a variety of cancers do not respond to retinoid therapy, while restoration of SMARCA4 expression restores retinoid sensitivity (Romero et al., 2012). Restoration of SMARCA1 *in vivo* significantly reduced lung cancer invasiveness and c-MYC expression, suggesting that inactivated SMARCA1 keeps cancer cells in an undifferentiated state and prevents its response to developmental and environmental stimuli (Romero et al., 2012). Conversely, in SH-SY5Y neuroblastoma cells, a commonly used model system of retinoid-dependent differentiation, attenuated expression of SMARCA1 prevented response to retinoids (Romero et al., 2012). Here, we show that elevated SMARCA4 expression corresponds strongly to poor outcomes for neuroblastoma patients, whereas elevated expression of SMARCA1 correlates to good outcome, suggesting antagonist roles in neuroblastoma for members of this gene family. SMARCA4 was also one of the genes identified as having protein-protein interactions with MYCN (mass spectrometry Co-IP). The NCI paediatric MATCH trial (NCT03155620) has recently detected SMARC mutations in relapsed neuroblastoma (Kumar et al., 2019), in keeping with the effectiveness of SMARCA inhibitors in strongly reducing the viability of the IMR32 relapsed cell line (Figure 2, compound 20). *In vitro* and *in vivo* loss-of-function experiments showed that SMARCA4 is essential for the proliferation of neuroblastoma cells (Jubierre et al., 2016). Taken together, SMARC inhibition provides a promising future therapeutic direction for relapsed MYCN amplified neuroblastoma.

Since few epigenetics drugs, are in clinical use for neuroblastoma, we pursued *in vitro* analyses to define novel potential therapeutics. IMR32, MYCN amplified neuroblastoma cells, showed broad susceptibility to epigenetic targeting compounds. Currently, 14 compounds within the SGC library are in clinical use or undergoing clinical trials (Supplementary Table 2), with five - Decitabine, Vorinostat Azacitidine, Olaparib and valproic acid, being tested in neuroblastoma. Given their advanced stage of testing elsewhere, we excluded these molecules from further characterisation. However, these agents are pan-antagonists that target DNMTs (Decitabine, Azacitidine), PARP (Olaparib) and HDACs (vorinostat and valproic acid), which usually results in numerous off-target effects, high toxicity and acquired resistance with prolonged drug exposure. Therefore, leads that have the potential to directly or indirectly modulate the activity of the MYCN oncogene are desirable. To identify such leads, we examined our omic datasets for genes targeted by SGC library compounds which significantly reduced IMR32 cell viability, which led to a primary focus on the CBP/p300 inhibitors: C646, I-CBP112 and CBP30.

The CBP and E1A Binding Protein P300 (known as EP300 or p300) proteins are closely related histone acetyltransferases (HATs) that act as transcriptional coactivators (Bannister and Kouzarides, 1996; Ogryzko et al., 1996). CBP/p300 inhibition can facilitate cellular reprogramming (Ebrahimi et al., 2019). Histone acetyltransferase inhibitors block SK-N-SH neuroblastoma cell growth *in vivo*, partly through CBP and p300 interactions (Gajer et al., 2015). Importantly, CBP/p300 also functions as a scaffold for various members of the transcriptional machinery and/or transcription factors (Barbieri et al., 2013). Among numerous partners forming transcriptional complexes, c-Myc binds to TATA-binding protein (TBP) and CBP, one half of the CBP/p300 coactivator complex, which has HAT activity and scaffolding functions (Patel et al., 2004). Thus, bound c-MYC together with CBP/p300 is localised to acetylated chromatin becoming a part of the transcriptional machinery to potentiate/modulate gene expression (He et al., 2013). CBP is also able to bind acetylated lysines on non-histone proteins, such as the p53 oncogene, and this interaction is required for the activation of the cyclin-dependent kinase inhibitor p21 (Mujtaba et al., 2004). It has also been shown that p300 is able to associate with c-Myc in mammalian cells through direct interactions with transactivation residues in c-MYC (N-terminal amino acids 1 to 110) and acetylates c-Myc protein. The resulting acetylation modulates the activity of c-MYC as well as the turnover of the protein (Faiola et al., 2005). Although highly similar in structure to c-MYC, there has been no study to reveal whether CBP/p300 co-factors are also able to modulate transcriptional activity and stability of the MYCN protein. Since CBP/p300 act as co-factors that facilitate localisation of transcription factors, among them MYC family members, modulating their activity represents a valuable and still poorly understood potential therapeutic approach.

Here, we show that MYCN amplified neuroblastoma cells are susceptible to C646 treatment, and that CBP targeting compounds should be further investigated as a single modality or combination therapeutic approach for high-risk neuroblastoma. C646 has been shown to increase survival in a mouse model of leukaemia (Wang et al., 2011), suggesting therapeutic utility across a range of cancer types. Since single agent treatments have thus far not provided the desired outcome benefit for high-risk neuroblastoma patients and since they can lead to acquired drug resistance, we also assessed the utility of a BRD4 inhibitor (JQ1) and CBP/p300 inhibitor (C646 and I-CBP112) combination. The combination treatments demonstrated that BRD and CBP/p300 inhibitors do not exert a synergistic effect, but rather a mild additive one. These compounds should continue to be assessed as single agents until more effective combination partners can be found for each. C646 was well tolerated in our differentiated neuron assay, Interestingly, the use of C646 has been reported in a study to impair memory formation during fear conditioning in mice, without reported adverse neurotoxic effects (Maddox et al., 2013).

## Conclusion

We reveal that MYCN amplified neuroblastoma cells are exquisitely sensitive to pharmaceutically induced epigenetic perturbations, with inhibition of numerous epigenetic mechanisms resulting in extensive loss of cell viability. We also show that MYCN interacts with the epigenetic machinery, both by protein-protein interactions, as well as, by directly regulating the expression of epigenetic modifiers. Thus, targeting of MYCN’s epigenetic network may prove an effective therapeutic avenue for high-risk neuroblastoma. In particular, the CBP/p300 inhibitor C646 shows promise as a targeted MYCN amplified neuroblastoma therapeutic.

## Materials and Methods

### Cell Culture and Treatments

For cell culture conditions and cell sources see Duffy et al. (2014). Briefly, the six neuroblastoma cell lines used were received as generous gifts from Dr. Frank Westermann (DKFZ, Heidelberg University, Germany) and Dr. Johannes Schulte (University Children’s Hospital Essen, Germany). The following lines were used: IMR-32 (IMR32) – ATCC code CCL-127, SH-SY5Y parental cell lines (PAR-SY5Y) (ATCC code CRL-2266), SMS-KCN (KCN)^5^, SMS-KCNR (KCNR)^6^, SH-SY5Y/6TR(EU)/pTrex-Dest-30/MYCN (SY5Y-MYCN) ^36,71^ – (generated in the Westermann Laboratory, DKFZ, Heidelberg). All NB cell lines were maintained in RPMI 1640 media (Gibco) supplemented with 10% fetal bovine serum (FBS) (Gibco) and 1% Penicillin-Streptomycin solution (Gibco). Transgenic cell line SY5Y-MYCN was maintained in the same media as for other cell lines and supplemented with G418 (0.2 mg/ml) (Sigma-Aldrich) and Blasticitidin (7.5 μg/ml) (Invitrogen). Induction of MYCN overexpression in SY5Y-MYCN cells was performed by adding Doxycycline hyclate (Sigma-Aldrich) at the final concentration of 1 μg/ml. Cells were cultivated in cell culture incubator (Thermo Scientific) at 37°C and 5% CO2. For differentiation assays and generation of neurons from NB cells, all-*trans* retinoic acid (RA) was added to growth media at the final concentration of 1 μM.

The SGC chemical library (Plate 9C – 45 compounds, Supplementary Table 2) was used for compound screening. Additional small molecules beyond the SGC library stocks used were from the following sources: C646 (SML0002, Sigma Aldrich), CBP30 (#4889, Tocris Bioscience), I-CBP112 (#4891, Tocris Bioscience), (+) -JQ1 (#87110, Selleck Chemicals), I-BET151 (ML0666, Sigma Aldrich) and all-*trans* Retinoic Acid (#R2625, Sigma Aldrich). Stock solutions were dissolved in DMSO. Compounds were replenished every 24 h for any treatment longer than a 24 h duration.

#### Cell Treatments

Doxycycline induction - SY5Y-MYCN cells were induced for MYCN expression by adding 1 μg/ml (final concentration) of Doxycycline to growth media. As the half-life of this drug in the culture media is approximately 48 h, growth media was replaced every day.

All-*trans* retinoic RA induction - For the generation of neurons from SH-SY5Y-MYCN cells, all-*trans* retinoic acid (RA) was added to growth media at the final concentration of 1 μM. RA was prepared in storage stocks (25 mg/ml) by dissolving the compound in DMSO. These stocks were stored at −80°C no longer than 6 months past the date of the stock preparation. Working stock (1 mM; 0.300044 mg/ml) was also kept at −80°C. Upon treatment, RA reagent and tissue culture dishes were kept protected from light.

Cell treatments with small molecule compounds - Cells were seeded in 96-well plates with 10,000 cells/well, with at least technical triplicates per treatment. Compounds were used in various concentrations, in accordance with experimental setup. In order to minimize the effect of compound degradation in the growth media, all compounds were replenished every 24 h for any treatment longer than the 24 h duration.

### Alamar Blue Cell Viability Assays

Alamar Blue assay (Resazurin sodium salt R7017, Sigma) provides the ability to monitor the combined effects of cellular health, apoptosis, proliferation, cell cycle function and control in a single assay. The active ingredient for Alamar Blue assay is resazurin which is water-soluble and stable in culture medium. Compared to other cell viability reagents it’s non-toxic and easily permeable through cell membranes. Measurements were performed in at least triplicate. Treatments were performed in 96-well plates. Upon the conclusion of treatments culture media was discarded and 0.1 ml of fresh RPMI-1640 medium with fresh Alamar Blue 0.05% stock (1:10 Alamar Blue to medium) was added directly to the wells. Cells were incubated for at least 3 h at 37°C and 5% CO2. After this time, absorbance was measured (Spectramax Plus384 Plate Reader, Molecular Devices accompanied with SoftMaxPro software) at excitation 570 nm and emission 600 nm. Calculations were performed in accordance with the manufacturer’s protocol^7^. The results are shown as the mean inhibition indices calculated by dividing each experimental result by the mean of the respective control values. For the washout experiments, media containing Alamar Blue was removed upon measurement and replaced with fresh media in order to facilitate the recovery of cells from the drug treatment.

#### Statistical Analysis

Statistical testing was performed in MS Excel. Data was tested for normal distribution (Shapiro-Wilk Test) using the online tool; https://www.statskingdom.com/320ShapiroWilk.html. Two sided t-tests with equal variance were performed by using the online tool; http://studentsttest.com/. Error bars are represented as plus/minus one standard deviation. Linear regression analysis was also performed in MS Excel (Pearson Correlation Coefficient test). IC_50_ calculation and dose response curve fitting were performed using the online tool, IC50 Toolkit^8^.

### Zebrafish Treatments

Zebrafish larvae (*Danio rerio*, AB and Tg[Fli1:EGFP] strains) were maintained on a 14h light/10h dark lighting cycle at 28.5°C. Drug treatments were conducted for 24 h (28 h post fertilisation [hpf]- 52 hpf), by waterborne exposure i.e., incubating the embryos in water containing epigenetic targeting compounds; 10 μM IBET-151, 5 μM C646, 10 μM CB112, 10 μM JQ1 and 10 μM CBP30. Embryos studies were approved by the UCD Animal Research Ethics Committee.

### Omic Datasets and Bioinformatics Tools

Ingenuity Pathway Analysis (IPA) software was also used for the ITR and pathway analysis. All survival curves were generated using the SEQC (Zhang et al., 2015) 498 neuroblastoma tumour dataset in the R2: Genomics Analysis and Visualization Platform^2^. Kaplan Meier curves were generated using the KaplanScan function that segregates a patient cohort in 2 groups on the basis of gene expression. The scanning function of this tool yields a cut-off where the difference in survival is most significant. KaplanScan was run with the following parameters: Type of Survival - event free; minimal groups size = 8. Multi-omic datasets (ChIP-seq, RNA-seq and interactome) were mined from published studies (Duffy et al., 2015, 2016; Harenza et al., 2017). Peaks were visualized using the SeqMonk analysis tool^9^ using default parameter settings. Briefly, using input ChIP-seq BAM files, sequencing probes were generated and peaks were called with MACS2, followed by annotation for the nearest gene. Distinct peaks were quantified and normalized against the largest dataset, while smoothing was performed using the pipeline for adjacent probes. Venn diagrams were generated using jvenn (Bardou et al., 2014)^10^. Epigenetic genes78 The dbEM, Database of Epigenetic Modifiers (Singh Nanda et al., 2016), was used for identifying MYCN’s epigenetic related targets^1^. Protein interaction networks were generated using the String database^11^.

## Data Availability Statement

The datasets presented in this study can be found in online repositories. The names of the repository/repositories and accession number(s) can be found below: ArrayExpress, www.ebi.ac.uk/arrayexpress, accession number E-MTAB-4100 (previously published, was mined for the current study).

## Ethics Statement

The animal study was reviewed and approved by The UCD Animal Research Ethics Committee.

## Author Contributions

AKr, WK, and DD designed and supervised the project. AKr, AKo, DD, and MH generated the data and performed data and bioinformatics analysis. UO and PC generated, designed and curated the epigenetic library. AKr and DD wrote the manuscript, with all authors (AKr, AKo, MH, UO, PC, WK, and DD) contributing to reading and editing the manuscript.

## Conflict of Interest

The authors declare that the research was conducted in the absence of any commercial or financial relationships that could be construed as a potential conflict of interest.

## Footnotes

1 http://crdd.osdd.net/raghava/dbem

2 http://r2.amc.nl

3 http://www.thesgc.org/

4 http://ic50.tk/index.html

5 http://www.cogcell.org/dl/NB_Data_Sheets/SMS-KCN_Cell_Line_Data_Sheet_COGcell_org.pdf

6 http://www.cogcell.org/dl/NB_Data_Sheets/SMS-KCNR_Cell_Line_Data_Sheet_COGcell_org.pdf

7 http://tools.lifetechnologies.com/content/sfs/manuals/PI-DAL1025-1100_TI%20alamarBlue%20Rev%201.1.pdf

8 http://ic50.tk/

9 https://www.bioinformatics.babraham.ac.uk/projects/seqmonk/

10 http://jvenn.toulouse.inra.fr/app/index.html

11 https://string-db.org/

## Supplementary Material

Supplementary Figure 1 Additional SMARC survival curves, MYC family gene expression and SGC library compound treatment cell viability results. **(A)** Kaplan-Meier survival curves showing the predictive strength of the expression levels of SMARC genes in neuroblastoma tumours on patient outcome. The SMARCs shown were predicted to be upstream regulators of MYCN’s genomic targets (ChIP-seq). SMARCA1and SMARCD3 was also a direct MYCN target (ChIP-seq). All survival curves were generated using the SEQC (Zhang et al., 2015) 498 neuroblastoma tumour dataset in the R2: Genomics Analysis and Visualization Platform (http://r2.amc.nl). **(B)** Gene expression levels of MYC family gene members in the IMR32 cell line, as assessed by RNA-seq (Duffy et al., 2015) **(C)** Cell viability assessment for IMR32 cells upon treatment with SGC library compounds. Compound dilutions are indicated above histograms (1x, 0.1x or 0.01x). Treatment bars – viability upon 48 h treatment. Washout bars – viability 72h upon washout, relative to corresponding untreated control at the same time point. Error bars indicate the mean ± SD.

Supplementary Figure 2 Additional expression and post-treatment imaging data. **(A)** CBP (CREBBP) transcript expression across the 39 neuroblastoma cell lines and two control non-neuroblastoma samples (human foetal brain tissue and a retinal pigment epithelial cell line, RPE1 cells) profiled by RNA-seq by Harenza et al. (2017), expression measured in FPKMBs. **(B)** Images of IMR32 cells after 48 h treatment with 1x dilution of epigenetic targeting compounds. Images were taken with 40x magnification. **(C)** Images of IMR32 cells after 72 h treatment with decitabine (5-aza-dC). **(D)** Kaplan-Meier survival curves showing the predictive strength of the expression level of SMYD2 in neuroblastoma tumours on patient outcome. Survival curve was generated using the SEQC (Zhang et al., 2015) 498 neuroblastoma tumour dataset in the R2: Genomics Analysis and Visualization Platform (http://r2.amc.nl).

Supplementary Figure 3 Additional network, outcome and viability data. **(A)** Protein-protein interaction network of the epigenetic MYCN interactome hits, and the MYCN protein, as generated by STRING (https://string-db.org/). Epigenetic hits were generated by overlapping our previously published MYCN mass spectrometry protein-protein interactome (Duffy et al., 2015, 2016) with epigenetic gene lists from Miremadi et al. (2007) and EpiDBase (Loharch et al., 2015) (Supplementary Table 1). **(B)** Kaplan-Meier survival curves showing the predictive strength of the expression levels of the CREB1, EP300 and BRD4 genes in neuroblastoma tumours on patient outcome. All survival curves were generated using the SEQC (Zhang et al., 2015) 498 neuroblastoma tumour dataset in the R2: Genomics Analysis and Visualization Platform (http://r2.amc.nl). **(C)** Log_10_ relative viability (Alamar Blue assay) upon 48 h combination CBP and BRD4 inhibitor treatment (C646 and JQ1) of MYCN amplified induced (Dox^+^) and non-induced (Dox^–^) SY5Y-MYCN cells. Error bars indicate the mean ± SD.

Supplementary Figure 4 Additional phenotypic screen data. **(A)** Zebrafish embryos treated for 24 h (28 h post fertilisation [hpf]- 52 hpf) with epigenetic targeting compounds (IBET-151, I-CB112 and CBP30). Magnification is 10x, images from 52 hpf. Melanocytes appear black due to their endogenous melanin. Otic vesicles (large black circles) mark the anterior pole (head) of the embryos.

## Abbreviations

ChIP-seq: Chromatin immunoprecipitation sequencing
CPMkb: read counts per million adjusted by gene length in kilobases
DE: differentially expressed
hpf: hours post fertilisation
IPA: Ingenuity pathway analysis
ITR: inferred transcriptional regulator
MNA: MYCN amplified
NB: neuroblastoma
TSS: transcription start site.
