# Supplementary figures and images for: A Chemo-Genomic Approach Identifies Diverse Epigenetic Therapeutic Vulnerabilities in MYCN-Amplified Neuroblastoma
Source: Front Cell Dev Biol. 2021 Apr 21;9:612518. doi: 10.3389/fcell.2021.612518 (PMC8097097; doi:10.3389/fcell.2021.612518)

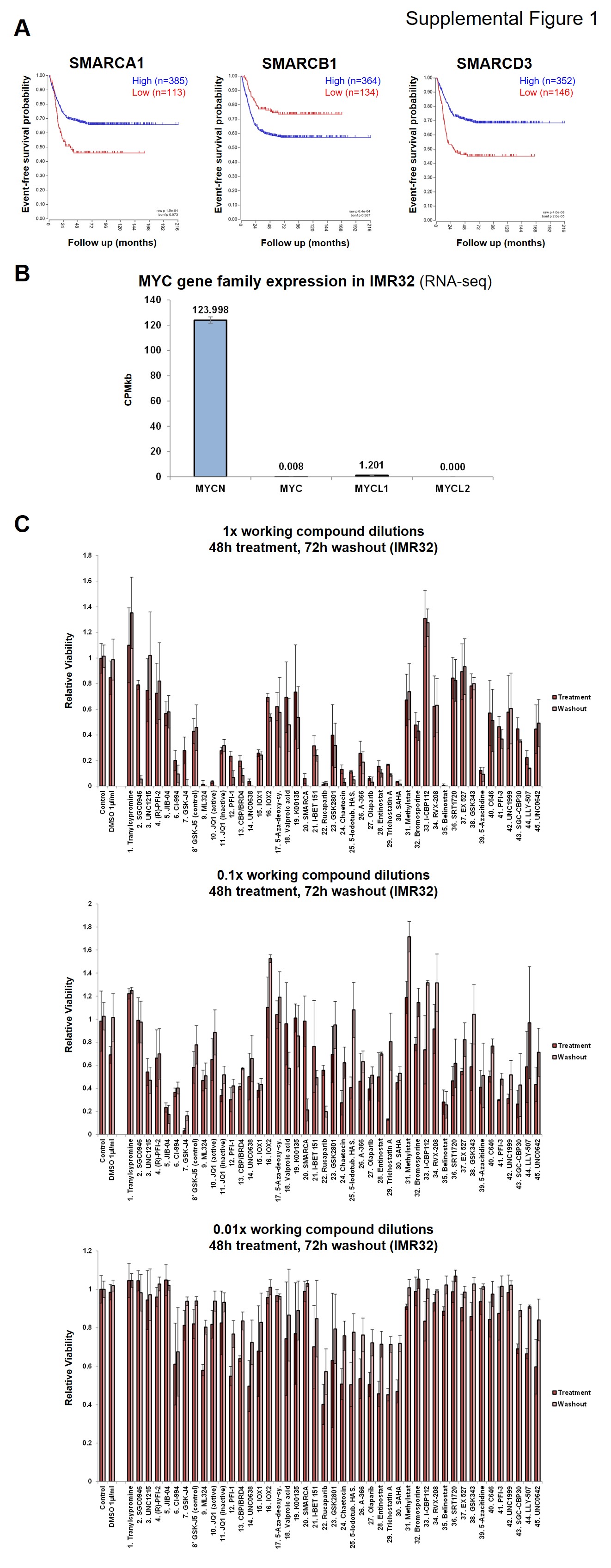

Supplement: Supplementary Figure 1 — Additional SMARC survival curves, MYC family gene expression and SGC library compound treatment cell viability results. (A) Kaplan-Meier survival curves showing the predictive strength of the expression levels of SMARC genes in neuroblastoma tumours on patient outcome. The SMARCs shown were predicted to be upstream regulators of MYCN’s genomic targets (ChIP-seq). SMARCA1and SMARCD3 was also a direct MYCN target (ChIP-seq). All survival curves were generated using the SEQC (Zhang et al., 2015) 498 neuroblastoma tumour dataset in the R2: Genomics Analysis and Visualization Platform (http://r2.amc.nl). (B) Gene expression levels of MYC family gene members in the IMR32 cell line, as assessed by RNA-seq (Duffy et al., 2015) (C) Cell viability assessment for IMR32 cells upon treatment with SGC library compounds. Compound dilutions are indicated above histograms (1x, 0.1x or 0.01x). Treatment bars – viability upon 48 h treatment. Washout bars – viability 72h upon washout, relative to corresponding untreated control at the same time point. Error bars indicate the mean ± SD. [file Image_1.JPEG]

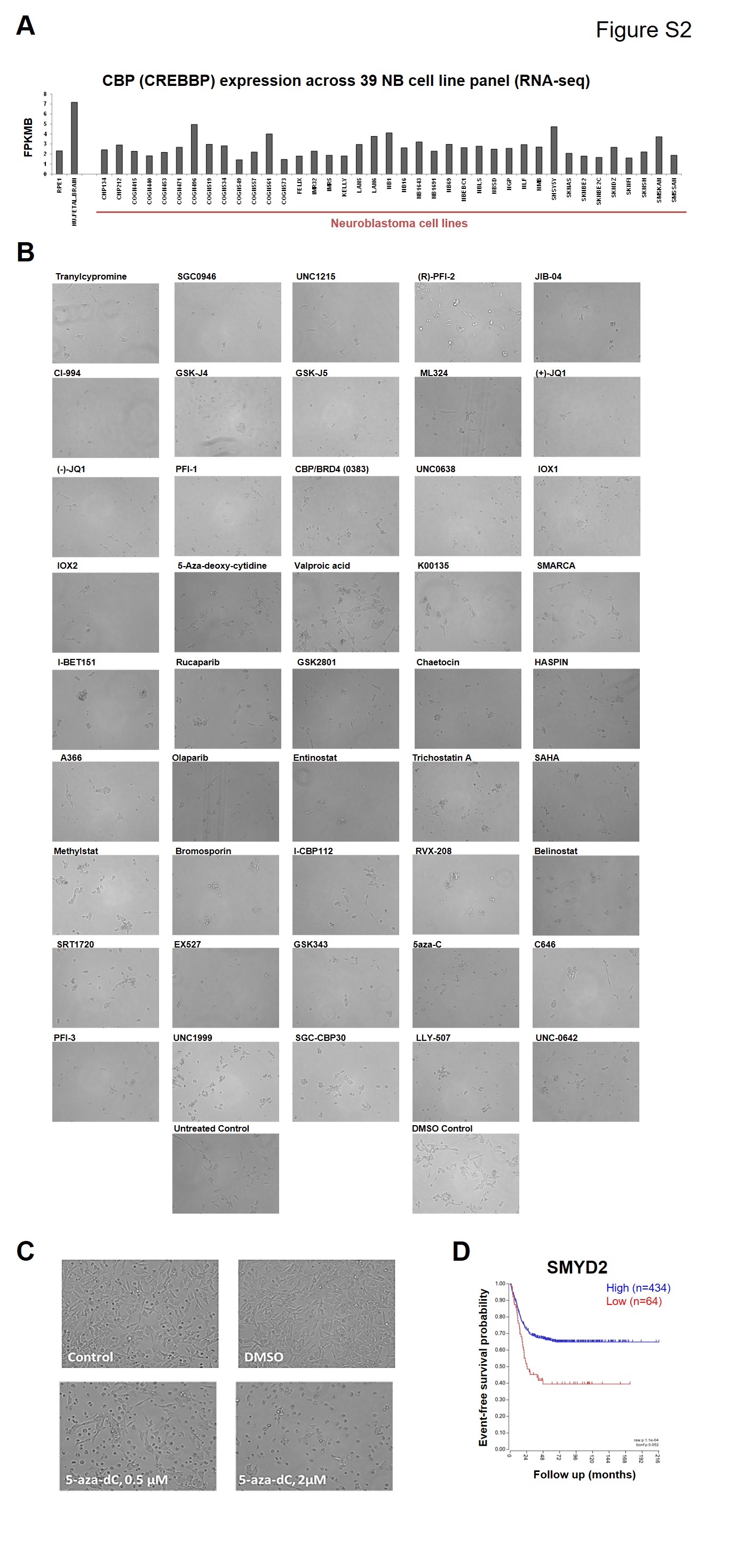

Supplement: Supplementary Figure 2 — Additional expression and post-treatment imaging data. (A) CBP (CREBBP) transcript expression across the 39 neuroblastoma cell lines and two control non-neuroblastoma samples (human foetal brain tissue and a retinal pigment epithelial cell line, RPE1 cells) profiled by RNA-seq by Harenza et al. (2017), expression measured in FPKMBs. (B) Images of IMR32 cells after 48 h treatment with 1x dilution of epigenetic targeting compounds. Images were taken with 40x magnification. (C) Images of IMR32 cells after 72 h treatment with decitabine (5-aza-dC). (D) Kaplan-Meier survival curves showing the predictive strength of the expression level of SMYD2 in neuroblastoma tumours on patient outcome. Survival curve was generated using the SEQC (Zhang et al., 2015) 498 neuroblastoma tumour dataset in the R2: Genomics Analysis and Visualization Platform (http://r2.amc.nl). [file Image_2.JPEG]

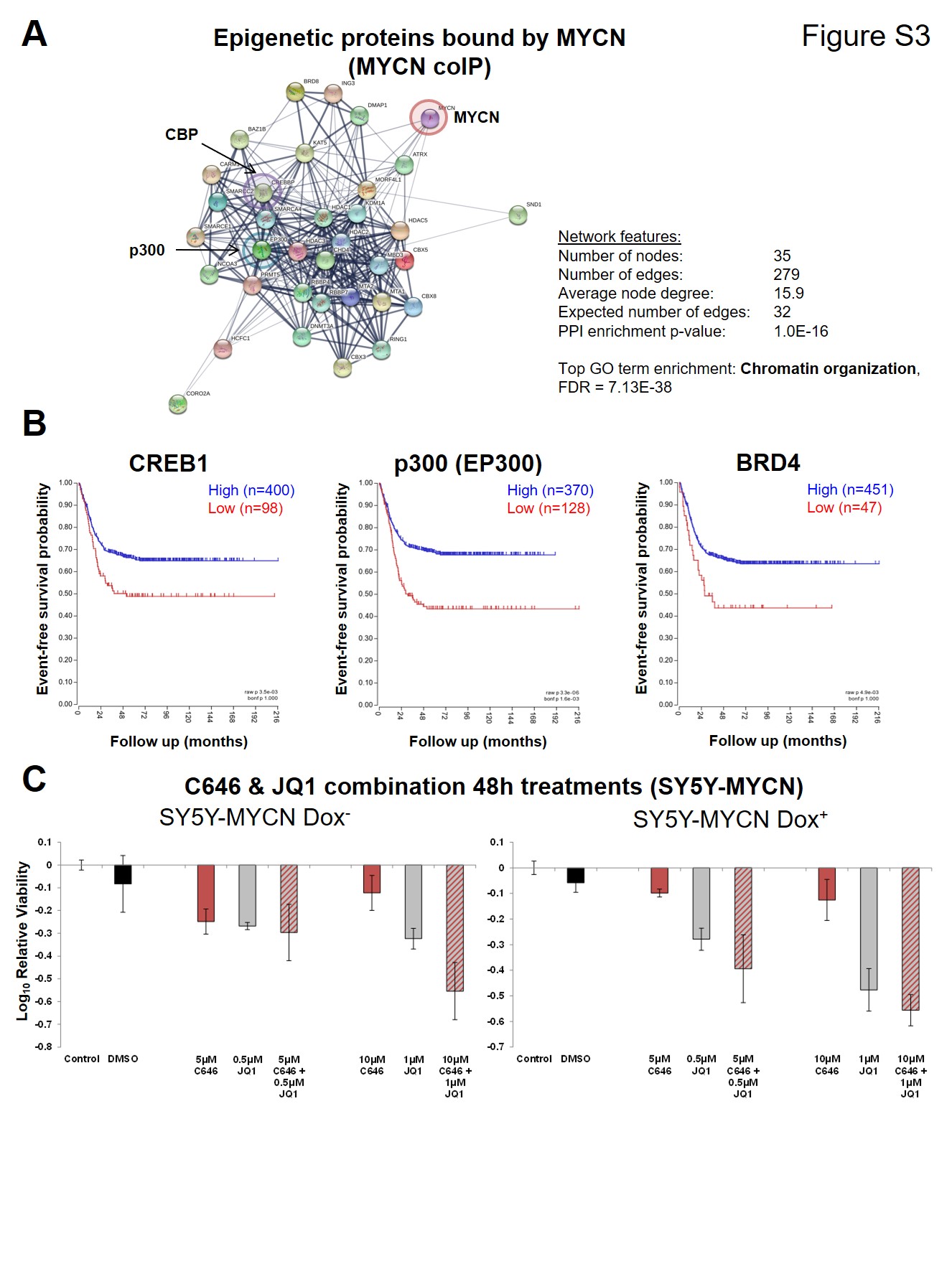

Supplement: Supplementary Figure 3 — Additional network, outcome and viability data. (A) Protein-protein interaction network of the epigenetic MYCN interactome hits, and the MYCN protein, as generated by STRING (https://string-db.org/). Epigenetic hits were generated by overlapping our previously published MYCN mass spectrometry protein-protein interactome (Duffy et al., 2015, 2016) with epigenetic gene lists from Miremadi et al. (2007) and EpiDBase (Loharch et al., 2015) (Supplementary Table 1). (B) Kaplan-Meier survival curves showing the predictive strength of the expression levels of the CREB1, EP300 and BRD4 genes in neuroblastoma tumours on patient outcome. All survival curves were generated using the SEQC (Zhang et al., 2015) 498 neuroblastoma tumour dataset in the R2: Genomics Analysis and Visualization Platform (http://r2.amc.nl). (C) Log10 relative viability (Alamar Blue assay) upon 48 h combination CBP and BRD4 inhibitor treatment (C646 and JQ1) of MYCN amplified induced (Dox+) and non-induced (Dox–) SY5Y-MYCN cells. Error bars indicate the mean ± SD. [file Image_3.JPEG]

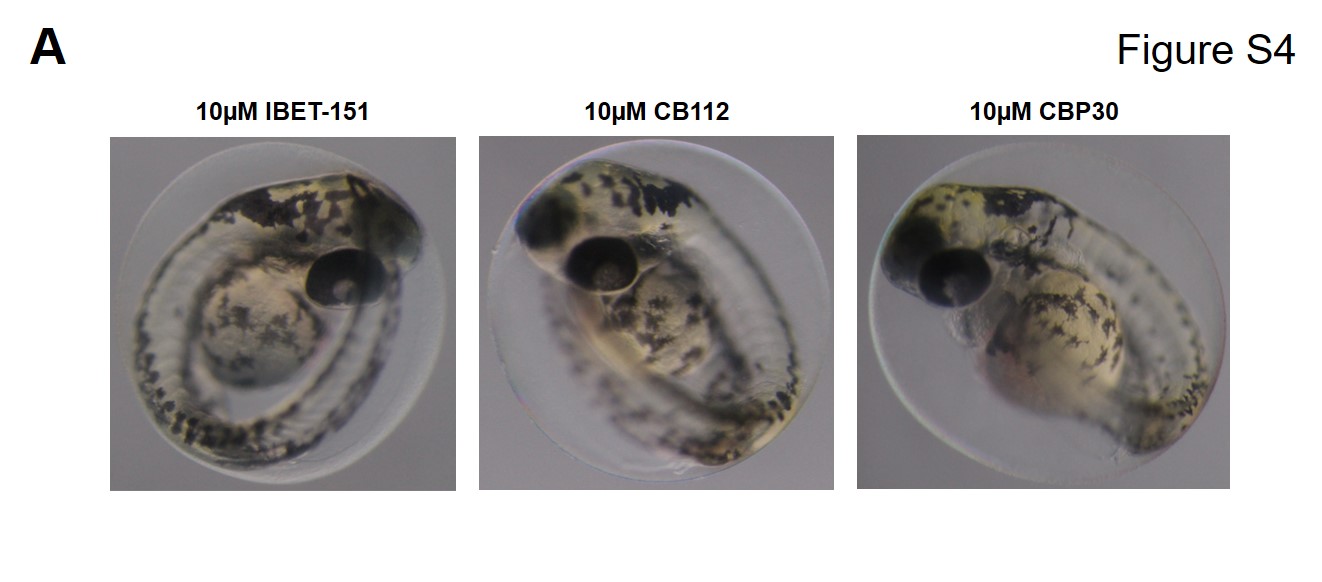

Supplement: Supplementary Figure 4 — Additional phenotypic screen data. (A) Zebrafish embryos treated for 24 h (28 h post fertilisation [hpf]- 52 hpf) with epigenetic targeting compounds (IBET-151, I-CB112 and CBP30). Magnification is 10x, images from 52 hpf. Melanocytes appear black due to their endogenous melanin. Otic vesicles (large black circles) mark the anterior pole (head) of the embryos. [file Image_4.JPEG]
