# Supplementary material for: A Chemo-Genomic Approach Identifies Diverse Epigenetic Therapeutic Vulnerabilities in MYCN-Amplified Neuroblastoma
Source: Front Cell Dev Biol. 2021 Apr 21;9:612518. doi: 10.3389/fcell.2021.612518 (PMC8097097; doi:10.3389/fcell.2021.612518)
Supplement: Supplementary file 5 [file Table_1.docx]

**Supplemental Table 1.** **Comparison of ChIP-seq MYCN target genes and mass spectrometry MYCN interactome datasets with curated epigenetic regulators lists**. Overlap with: epigenetic gene list from Miremadi et al. (Miremadi et al. 2007) (89 genes), EpiDBase (Loharch et al. 2015) (220 genes) and our own MYCN mass spectrometry protein-protein interactome (Duffy et al. 2015; Duffy et al. 2016). CBP and HDAC2 (highlighted in blue font) are MYCN ChIP-seq targets molecules common to all three lists, while HDAC9, PRDM1, SMYD3 and CBX8 (highlighted in red font) are common to least two lists.

| **Miremadi et al. & ChIP-seq** | **EpiDBase & ChIP-seq** | | | **EpiDBase & MYCN MS-IP** | | |
| --- | --- | --- | --- | --- | --- | --- |
| CDYL | ANKRD28 | KDM4C | PRKAB2 | ATRX | HDAC1 | RBBP7 |
| **CBP (CREBBP)** | BARD1 | KDM6A | PRKAG2 | BAZ1B | **HDAC2** | RING1 |
| HAT1 | BAZ2B | L3MBTL3 | PRKCA | BRD8 | HDAC3 | SMARCA4 |
| **HDAC2** | CBX4 | L3MBTL4 | PRMT6 | CARM1 | HDAC5 | SMARCC2 |
| **HDAC9** | **CBX8** | LIMK2 | RPS6KA3 | CBX3 | ING3 | SMARCE1 |
| MBD2 | CDK8 | MYSM1 | RPS6KA5 | CBX5 | KAT5 | SND1 |
| MLL3 | CHUK | NEK6 | SETMAR | **CBX8** | KDM1A |  |
| NCOA1 | **CBP(CREBBP)** | PPP1CC | SMYD2 | CHD4 | MBD3 |  |
| **PRDM1** | DAPK1 | PPP2R2B | **SMYD3** | CORO2A | MORF4L1 |  |
| PRDM2 | EYA1 | PPP2R2C | SUV420H2 | **CBP (CREBBP)** | MTA1 |  |
| **SMYD3** | **HDAC2** | PPP3CA | TAF1 | DMAP1 | MTA2 |  |
|  | HDAC4 | PPP5C | UBE2H | DNMT3A | NCOA3 |  |
|  | **HDAC9** | PPP6R3 | UBR2 | EP300 | PRMT5 |  |
|  | KDM2A | **PRDM1** | USP3 | HCFC1 | RBBP4 |  |
| **11/89 genes** | **42/220 genes** | | | **34/220 genes** | | |

**References:**

Duffy, D.J., Krstic, A., Halasz, M., Schwarzl, T., Fey, D., Iljin, K., et al. (2015). Integrative omics reveals MYCN as a global suppressor of cellular signalling and enables network-based therapeutic target discovery in neuroblastoma. *Oncotarget* 6(41)**,** 43182-43201. doi: 10.18632/oncotarget.6568.

Duffy, D.J., Krstic, A., Schwarzl, T., Halasz, M., Iljin, K., Fey, D., et al. (2016). Wnt signalling is a bi-directional vulnerability of cancer cells. *Oncotarget* 7(37)**,** 60310-60331.

Harenza, J. L., Diamond, M. A., Adams, R. N., Song, M. M., Davidson, H. L., Hart, L. S., et al. (2017). Transcriptomic profiling of 39 commonly-used neuroblastoma cell lines. *Sci. Data* 4:170033. doi: 10.1038/sdata.2017.33

Loharch, S., Bhutani, I., Jain, K., Gupta, P., Sahoo, D.K., and Parkesh, R. (2015). EpiDBase: a manually curated database for small molecule modulators of epigenetic landscape. *Database* 2015.

Miremadi, A., Oestergaard, M.Z., Pharoah, P.D., and Caldas, C. (2007). Cancer genetics of epigenetic genes. *Human molecular genetics* 16(R1)**,** R28-R49.
