# Supplementary material for: A Chemo-Genomic Approach Identifies Diverse Epigenetic Therapeutic Vulnerabilities in MYCN-Amplified Neuroblastoma
Source: Front Cell Dev Biol. 2021 Apr 21;9:612518. doi: 10.3389/fcell.2021.612518 (PMC8097097; doi:10.3389/fcell.2021.612518)
Supplement: Supplementary file 6 [file Table_2.DOCX]

| No. | Compound | Class - Target | Stock (mM)  (1,000x working stock) | PubChem ID | ClinicalTrials.gov Identifier |
| --- | --- | --- | --- | --- | --- |
| 1 | Tranylcypromine | Lysine demethylases - LSD1 | 20 | 19493 | NCT02261779 |
| 2 | SGC0946 (DOT1L probe) | Histone methyltransferase - DOT1L | 7.5 | 71729976 | no |
| 3 | UNC1215 (L3MBTL3 probe) | Methyl Lysine Binder - L3MBTL3 | 5 | 57339144 | no |
| 4 | (R)-PFI-2 ((SETD7 probe) | Histone methyltransferase - SETD7 | 2 | 71300326 | no |
| 5 | JIB-04 | Histone demethylase - Pan JmjC | 0.05 | 6519698 | no |
| 6 | CI-994 | HDAC - 1, 2, 3, (8) | 1 | 2746 | NCT00005624, NCT00005093, NCT00004861 |
| 7 | GSK-J4 (KDM probe) | Lysine demethylases - JMJD3, UTX, JARID1B | 10 | 1729975 | no |
| 8 | GSK-J5 (control) | Lysine demethylases - Negative control | 10 | 126456082 | no |
| 9 | ML324 | Histone demethylase - JMJD2E | 5 | 44143209 | no |
| 10 | (+)-JQ1 (active enantiomer) | Bromodomains - BRD2, BRD3, BRD4, BRDT (BET) | 0.1 | 46907787 | TEN-01 Analogue - NCT02308761, NCT01987362 |
| 11 | (-)-JQ1 (inactive stereoisomer) | Bromodomains - Negative control | 0.1 | 49871818 | no |
| 12 | PFI-1 (BET probe) | Bromodomains - BRD2, BRD3, BRD4, BRDT (BET) | 5 | 71271629 | no |
| 13 | CBP/BRD4 (0383) | Bromodomains - CBP, BRD4(1) | 5 | in development | no |
| 14 | UNC0638 (G9a probe) | Histone methyltransferase - G9a, GLP | 1 | 46224516 | no |
| 15 | IOX1 (5COOH-8HQ) | Lysine demethylases - pan-2-OG | 40 | 459617 | no |
| 16 | IOX2 (PHD2 probe) | Prolyl-Hydroxylases - PHD2 (EGLN1) | 10 | 54685215 | no |
| 17 | 5-Aza-deoxy-cytidine | DNA methyltransferase (DNMT) - DNMT1/3 | 5 | 451668 | NCT01241162 (NB), NCT00075634 (NB), NCT01546038, NCT03236857 |
| 18 | Valproic acid | HDAC - aliphatic acid compounds | 1000 | 3121 | NCT01204450 (NB) |
| 19 | K00135 | Kinase inhibitor - ATP competitive - PIM | 1 | 5459373 | no |
| 20 | SMARCA | Bromodomains - SMARCA, PB1 | 2.5 | in development | no |
| 21 | I-BET 151 (BET probe) | Bromodomains - BRD2/3/4 | 1 | 52912189 | no |
| 22 | Rucaparib | Poly ADP ribose polymerase (PARP) | 10 | 9931954 | 44 studies active/recruiting, no NB |
| 23 | GSK2801 (BAZ2B/A probe) | Bromodomains - BAZ2A, BAZ2B | 1 | 73010930 | no |
| 24 | Chaetocin | Histone methyltransferase - SUV39H1 | 0.05 | 161591 | no |
| 25 | 5-Iodotubercidin HASPIN | Kinase inhibitor - ATP mimetic - Haspin | 1 | 97297 | no |
| 26 | A-366 (G9a/GLP probe) | Histone methyltransferase - G9a, GLP | 2 | 76285486 | no |
| 27 | Olaparib | Poly ADP ribose polymerase (PARP) | 1 | 23725625 | NCT03233204 (NB), NCT03155620 (NB) |
| 28 | Entinostat (MS-275) | HDAC - ortho-amino anilides | 0.5 | 4261 | 29 studies active/recruiting, no NB |
| 29 | Trichostatin A | HDAC - hydroxamic acids - Class I & II | 0.5 | 444732 | 15 studies active/recruiting, no NB |
| 30 | SAHA (Vorinostat) | HDAC - hydroxamic acids | 1 | 5311 | NCT03332667 (NB), NCT02035137 (NB), NCT02559778 (NB), NCT04308330 (NB) |
| 31 | Methylstat | Histone demethylase | 0.5 | 53392493 | no |
| 32 | Bromosporin | Bromodomains - pan-Bromodomain | 1 | 72943187 | no |
| 33 | I-CBP112 (CREBBP/EP300 probe) | Bromodomains - CREBBP, EP300 | 1 | 90488984 | no |
| 34 | RVX-208 | Bromodomains - BRD2, BRD3, BRD4, BRDT (BET, BD2) | 5 | 24871506 | 9 studies, no NB |
| 35 | Belinostat | HDAC - hydroxamic acids | 5 | 6918638 | 5 studies active/recruiting, no NB |
| 36 | SRT1720 | HDAC - SIRT1 (indirect?) activator | 1 | 5232708 | no |
| 37 | EX 527 | HDAC - SIRT1 | 1 | 5113032 | NCT04184323 |
| 38 | GSK343 (EZH2 probe) | Histone methyltransferase - EZH2 | 3 | 71268957 | no |
| 39 | 5-Azacitidine | DNA methyltransferase (DNMT) | 10 | 9444 | NCT03236857 (NB) |
| 40 | C646 | Histone acetyltransferase (HAT) p300/CBP | 1 | 1285941 | no |
| 41 | PFI-3 | Bromodomains - SMARCA2/4, PB1(5) | 1 | 78243717 | no |
| 42 | UNC1999 (EZH2/1 probe) | Histone methyltransferase - EZH2 | 1 | 72551585 | no |
| 43 | SGC-CBP30 (CREBBP/EP300 probe) | Bromodomains - CREBBP, EP300 | 1 | 72201027 | no |
| 44 | LLY-507 | Histone methyltransferase - SMYD2 | 1 | 91623361 | no |
| 45 | UNC0642 (G9a/GLP probe) | Histone methyltransferase - G9a, GLP | 1 | 53315878 | no |
